# Theta-Burst Stimulation of Hippocampal Slices Induces Network-Level Calcium Oscillations and Activates Analogous Gene Transcription to Spatial Learning

**DOI:** 10.1371/journal.pone.0100546

**Published:** 2014-06-20

**Authors:** Graham K. Sheridan, Emad Moeendarbary, Mark Pickering, John J. O'Connor, Keith J. Murphy

**Affiliations:** 1 Department of Physiology, Development and Neuroscience, University of Cambridge, Cambridge, United Kingdom; 2 Hughes Hall, University of Cambridge, Cambridge, United Kingdom; 3 School of Medicine and Medical Science, Health Sciences Centre, University College Dublin, Dublin, Ireland; 4 UCD School of Biomolecular and Biomedical Science, Conway Institute, University College Dublin, Dublin, Ireland; 5 Neurotherapeutics Research Group, UCD School of Biomolecular and Biomedical Science, Conway Institute, University College Dublin, Dublin, Ireland; Univ. Kentucky, United States of America

## Abstract

Over four decades ago, it was discovered that high-frequency stimulation of the dentate gyrus induces long-term potentiation (LTP) of synaptic transmission. LTP is believed to underlie how we process and code external stimuli before converting it to salient information that we store as 'memories'. It has been shown that rats performing spatial learning tasks display theta-frequency (3–12 Hz) hippocampal neural activity. Moreover, administering theta-burst stimulation (TBS) to hippocampal slices can induce LTP. TBS triggers a sustained rise in intracellular calcium [Ca^2+^]_i_ in neurons leading to new protein synthesis important for LTP maintenance. In this study, we measured TBS-induced [Ca^2+^]_i_ oscillations in thousands of cells at increasing distances from the source of stimulation. Following TBS, a calcium wave propagates radially with an average speed of 5.2 µm/s and triggers multiple and regular [Ca^2+^]_i_ oscillations in the hippocampus. Interestingly, the number and frequency of [Ca^2+^]_i_ fluctuations post-TBS increased with respect to distance from the electrode. During the post-tetanic phase, 18% of cells exhibited 3 peaks in [Ca^2+^]_i_ with a frequency of 17 mHz, whereas 2.3% of cells distributed further from the electrode displayed 8 [Ca^2+^]_i_ oscillations at 33 mHz. We suggest that these observed [Ca^2+^]_i_ oscillations could lead to activation of transcription factors involved in synaptic plasticity. In particular, the transcription factor, NF-κB, has been implicated in memory formation and is up-regulated after LTP induction. We measured increased activation of NF-κB 30 min post-TBS in CA1 pyramidal cells and also observed similar temporal up-regulation of NF-κB levels in CA1 neurons following water maze training in rats. Therefore, TBS of hippocampal slice cultures *in vitro* can mimic the cell type-specific up-regulations in activated NF-κB following spatial learning *in vivo*. This indicates that TBS may induce similar transcriptional changes to spatial learning and that TBS-triggered [Ca^2+^]_i_ oscillations could activate memory-associated gene expression.

## Introduction

The molecular and cellular mechanisms that facilitate learning and memory formation have been studied intensely for decades. In order to understand these complex cognitive processes, neuroscientists have developed ‘simple’ *in vitro* reductionist approaches that model the cellular basis of memory formation. Since the discovery by Bliss and Lømo [1] that high-frequency electrical stimulation (HFS) can lead to a long-lasting enhancement of synaptic transmission, it has been suggested that this experimentally-induced phenomenon, known as long-term potentiation (LTP), can recapitulate many of the molecular and cellular processes that facilitate memory formation [2]–[6]. Both LTP and memory formation have been shown to depend on new protein synthesis within stimulated cells [7]–[9]. Transcription factors such as cyclic AMP-responsive element binding protein (CREB) and nuclear factor-kappa B (NF-κB) are reported to play crucial roles in the gene transcription that drives enhanced synaptic transmission [10]–[18]. Upstream of gene transcription, however, are a multitude of intracellular signalling cascades involving several key protein kinases, including protein kinase A (PKA) and calcium calmodulin-dependent kinase II (CaMKII) [19]. Activation of these kinases is intimately linked to elevations in intracellular calcium concentration [20], [21]. Therefore, calcium acts as a second messenger and initiates intracellular signalling that promotes transcription factor translocation to the nucleus and new protein synthesis required for LTP maintenance [22], [23].

The role of calcium in LTP induction and maintenance is complex. Pre-synaptic glutamate release can activate post-synaptic AMPA receptors causing a depolarisation-induced unblocking of NMDA receptors which allows calcium influx to the post-synaptic cell [2], [24]. Large increases in intracellular calcium concentration [Ca^2+^]_i_ can lead to the opening of voltage-dependent calcium channels (VDCC). Elevations in [Ca^2+^]_i_ can also promote calcium-induced calcium release (CICR) from intracellular stores [25]. Repeated activation of intracellular stores can produce sustained oscillations in [Ca^2+^]_i_ [26], [27]. Because calcium is a ubiquitous second messenger and is involved in translating many extracellular stimuli into intracellular biochemical reactions, the precise dynamics of the calcium response may act as a coded message that allows the cell to decipher the initial stimulus. Therefore, the amount of extracellular calcium [Ca^2+^]_o_ that enters the cell and how fast this occurs could influence the intracellular signalling cascades that are activated. Similarly, the time it takes for elevated [Ca^2+^]_i_ to return to basal levels and whether intracellular calcium oscillations are initiated will influence the physiological response of the cell [28].

Theta-burst electrical stimulation mimics *in vivo* firing frequencies (3–12 Hz) in the CA1 region of the hippocampus of rats performing a spatial learning task [29]–[31]. Moreover, theta-burst stimulation (TBS) is arguably the most effective pattern of activity for LTP induction [31]–[33]. The TBS protocol used in this study has been shown to activate both NMDA receptors and VDCCs to produce a robust and long-lasting form of LTP [34]–[37]. To our knowledge, however, simultaneous calcium imaging of hundreds of individual cells in response to theta-burst stimulation has not yet been described in detail. There are many elegant studies that have concentrated on characterising the calcium response of individual synapses or small numbers of cells close to the stimulation site [38]–[43]. Therefore, not much is known about the network-level effects of TBS on [Ca^2+^]_i_ fluctuations at large distances from the site of electrical stimulation. Our aim, in this study, was to measure and characterise single-cell calcium responses to TBS in a large proportion of a hippocampal slice. We analysed the calcium dynamics of 6,536 cells located within 500 µm from the point of stimulation. TBS triggers an initial radially-propagating calcium wave that decays exponentially in intensity with respect to distance from the electrode. Moreover, this first wave induces multiple and regular calcium oscillations in the hippocampal cellular network.

It has been shown that protein kinase activation is sensitive to intracellular calcium oscillations [44], [45] and CaMKII, for example, has been shown to activate the transcription factor, NF-κB, following glutamate-induced synaptic transmission [46]. Interestingly, it has also been reported that different [Ca^2+^]_i_ oscillation frequencies can preferentially activate different transcription factors, with low frequency [Ca^2+^]_i_ oscillations preferentially activating NF-κB over NF-AT and Oct/OAP transcription factor gene expression [47]. We, therefore, monitored changes in NF-κB activation 30 min after TBS in the three main neuronal populations of the hippocampus, i.e. dentate gyrus, CA3 and CA1. We report increased NF-κB activation in the CA1 region after TBS. More importantly, we also found the same temporal up-regulation in NF-κB activation in CA1 neurons in rats trained in the Morris water maze spatial learning task. These similar temporal and cell type-specific activations of NF-κB following both learning and TBS suggest that NF-κB is mobilised to a common goal in these two models of synaptic plasticity [48]–[50]. Moreover, the precise frequency of the sustained calcium oscillations in the hippocampal network, triggered by TBS, may be responsible for activating specific gene expression changes important to both LTP maintenance and to memory formation.

## Materials and Methods

### Organotypic hippocampal cultures

Organotypic hippocampal cultures were prepared according to Stoppini et al. [51]. Briefly, post-natal day 7 male Wistar rats were decapitated without anaesthetic and their brains quickly dissected out and placed into ice-cold Earle's balanced salt solution (EBSS) for 1 min. Both hippocampi were dissected and cut into 400 µm slices using a McIlwain tissue chopper. Slices were separated and arranged onto organotypic inserts (3 per insert, Millicell PICMORG50). The inserts were housed in standard 6-well cell culture plates which were kept in an incubator at 35°C and 5% CO_2_. The slices were grown using an interface method with 1 ml organotypic medium supplying the undersurface of the slice. The organotypic medium consisted of 50% minimum essential medium (MEM, Gibco), 25% EBSS (Gibco), 25% heat-inactivated horse serum (Sigma) and supplemented with 2 mM glutamine, 28 mM D-glucose, 100 U/ml penicillin/streptomycin and 25 mM HEPES. The first medium change was conducted 24 h following slice preparation with subsequent medium changes occurring every 2 days. Slices were maintained for 21 days *in-vitro* (DIV) prior to experimentation.

### Calcium imaging and theta-burst stimulation of organotypic hippocampal cultures

**Figure 1:**
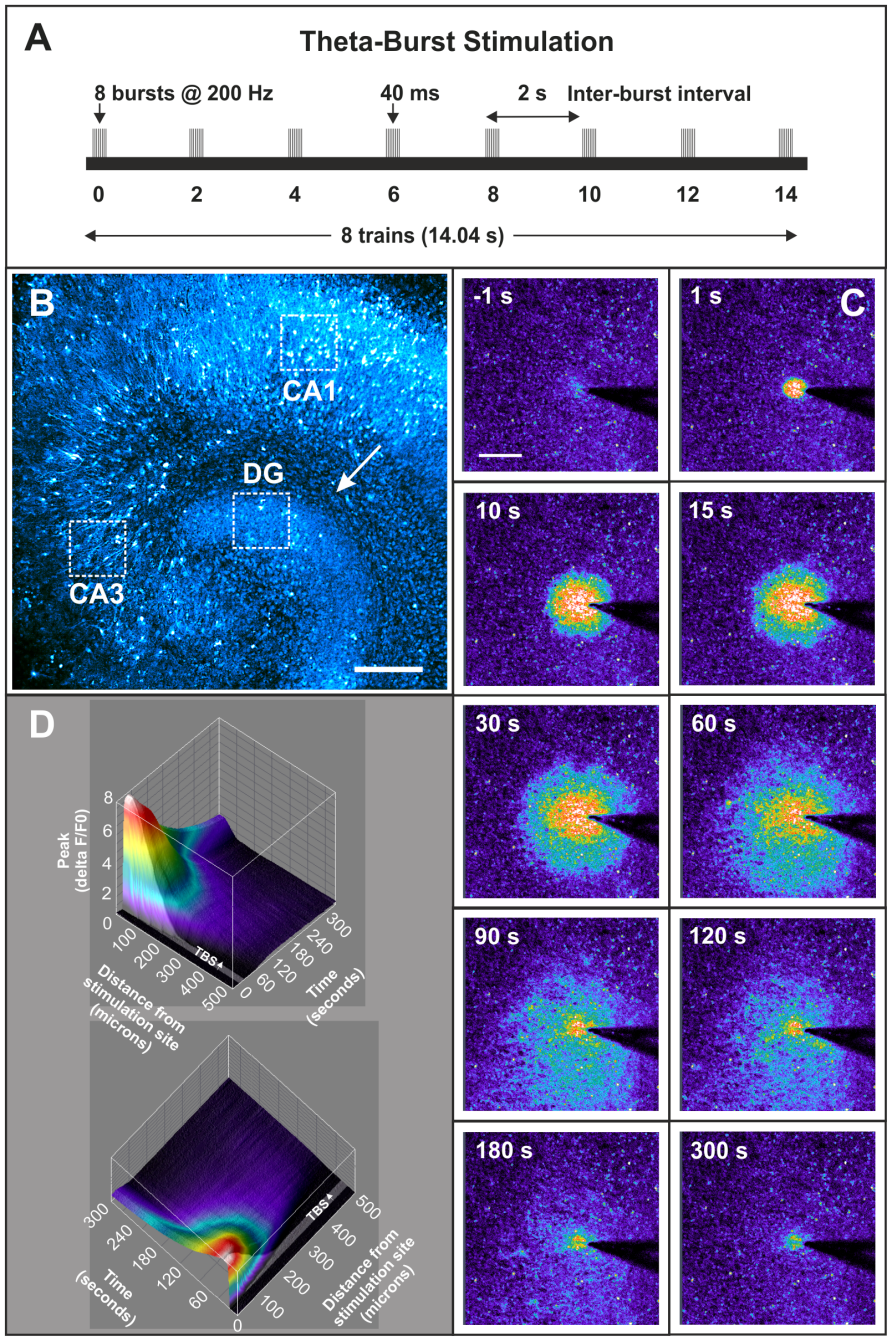
Theta-burst stimulation of hippocampal slice cultures. (A) Schematic representation of the theta-burst stimulation (TBS) protocol used. TBS consisted of 8 trains with 8 bursts in each train (i.e. 64 stimuli in total). The train duration was 40 ms making the burst frequency 200 Hz and the inter-burst interval was 2 s. Therefore, TBS took 14.04 s to complete. (B) 21 day *in vitro* (DIV) organotypic hippocampal culture loaded with Fluo-4 AM calcium indicator. A pseudo-colour fluorescence intensity palette (cyan → white) indicates levels of lowest to highest basal calcium concentration in the slice culture. The arrow indicates the point of electrical stimulation in the molecular layer of the dentate gyrus. The white boxes represent the areas imaged and analysed for changes in nuclear NF-κB activation. Scale bar = 200 µm. (C) Low-magnification (10X) images representing the calcium response at various time-points post theta-burst stimulation (TBS). A pseudo-colour rainbow palette (black/violet → red/white) is used to illustrate the changes in calcium levels more clearly, i.e. the ‘hotter’ the colour, the higher the relative intracellular calcium concentration. The first image represents baseline calcium levels 1 s before TBS began. TBS lasts for 14.04 s but the calcium wave continues to propagate in a radial fashion after TBS has terminated. Scale bar = 200 µm. (D) A four-dimensional surface plot representing the changes in intracellular calcium levels in a 21 DIV organotypic hippocampal slice culture in response to TBS to the molecular layer of the dentate gyrus. The X-axis represents time, i.e. 20 s baseline followed by a 14.04 s shaded area representing the period of TBS, followed by a 4 min 56 s period (296 s) post-TBS. Graphed along the Y-axis is distance from the electrode tip, i.e. 0–500 µm. The Z-axis represents the peak calcium response (i.e. ΔF/F_0_). Also, mapped onto the surface plot is the ‘time-to-peak’ data which is represented by the rainbow palette, i.e. the ‘hotter’ the colour, the faster the cell achieved its peak calcium level in response to TBS.

21 DIV organotypic hippocampal cultures were prepared for calcium imaging experiments by transferring inserts to room-temperature BSS (buffered salt solution) composed of 130 mM NaCl, 5.4 mM KCl, 1.8 mM CaCl_2_, 2 mM MgSO_4_, 5.5 mM D-glucose and 20 mM HEPES, pH 7.3. The insert membranes were cut using a scalpel and individual slices were transferred to 35 mm Petri dishes containing 2 ml of 3 µM fluo-4 AM calcium indicator (Molecular Probes) in BSS and allowed to incubate in the dark for 30 min. Following ester loading of the calcium dye, slices were transferred to a custom-built chamber containing fresh BSS to be imaged using the 10X/0.3W Ph1 water-immersion lens on a LSM 5 Pascal confocal microscope (Zeiss). All experiments were conducted at room temperature. A stimulating monopolar glass electrode was lowered onto the surface of the slice and positioned in the molecular layer of the dentate gyrus. The scanning laser on the microscope was programmed to capture 1 frame per second for 330 s allowing a baseline recording of 20 s before a theta-burst stimulus protocol of 10 volts in magnitude was delivered to the tissue (Figure 1). The electrode used was pulled from a borosilicate glass capillary (GC150F-10, Harvard Apparatus, USA) using a P-97 Brown-Flaming micropipette puller (Sutter Instruments Co.) and had a tip diameter of 4 µm and a resistance of approx. 1.5 MΩ for monopolar stimulation of organotypic slice cultures. The same stimulating electrode was used for each slice in order to minimise any inherent slice-to-slice variability in input/output relationships evoked by the stimulus voltage (Grass Instruments SIU5 stimulus isolation unit, 10 V output). Moreover, the magnitude, velocity and propagation distance of the TBS-induced calcium wave was similar in all slices tested. TBS consisted of 8 trains with an inter-burst interval of 2 s. Each train lasted 40 ms and consisted of 8 bursts at 200 Hz. Therefore, TBS was complete after 14.04 s (Figure 1A). The calcium response to TBS was visualized and recorded over a 5 min 10 s period. Slices were then transferred back into the incubator in 1 ml of fresh pre-equilibrated organotypic medium for 30 min post-TBS. Control slices received the exact same treatment minus theta-burst stimulation. A total of 15 slices were stimulated using this TBS protocol. Three control and three stimulated slices were chosen at random for immunofluorescent labelling of activated NF-κB.

### Immunofluorescent staining of organotypic hippocampal cultures

Organotypic hippocampal cultures were prepared for immunofluorescence according to Gogolla et al. [52]. Briefly, slices were removed from the incubator and received two 30 s washes in phosphate buffered saline (PBS) before being fixed in an ice-cold solution of 4% formaldehyde for 5 min. Slices received another 1 min wash in PBS and were transferred to a solution of 20% methanol for 3 min. Slices were again washed in PBS for 5 min and transferred to a 0.5% solution of Triton X. The slices were permeabilized in this solution for 18 h at 4°C. They were then washed in PBS and transferred for 4 h into a blocking solution consisting of 20% BSA (bovine serum albumin) in PBS. Following this blocking step, slices were incubated with the primary antibody MAB3026 (Chemicon) for 18 h at 4°C in a solution containing 5% BSA. This antibody binds to an epitope overlapping the nuclear location signal on the p65 subunit of the NF-κB heterodimer. Thus, it selectively binds to the activated form of NF-κB. The slices were then washed and incubated with an anti-mouse secondary antibody labelled with Cy3 (Jackson ImmunoResearch) for 4 h at room temperature. Nuclei were counterstained using Hoechst 33258 (Molecular Probes). Slices were placed on microscope slides and mounted with coverslips using OCT (optimal cutting temperature compound, Agar Scientific) and sealed with clear nail varnish. Slides were stored in the dark at room temperature before being imaged.

### Animal care and behavioural assessment

Postnatal day 80 male Wistar rats (330–380 g) were obtained from the Biomedical Facility at University College Dublin, Ireland. All experimental procedures were approved by the Animal Research Ethics Committee of the Biomedical Facility at UCD and were carried out by individuals who held the appropriate license issued by the Minister for Health and Children. Animals were socially housed in groups of 4 and given *ad libitum* access to food and water. The experimental room was kept on a 12 h light/dark cycle at 22±2°C. The animals' behaviour was assessed in an open field apparatus (620 mm long, 620 mm wide and 150 mm high) both 48 h and 24 h prior to commencement of training. All rats behaved normally in the open field apparatus. The base of the open field box was demarcated into an 8×8 grid. The rat was placed in the centre of the box and its locomotion was monitored by counting the number of squares crossed in a 5 min period. The number of times the rats reared on their hind legs was also noted. Behavioural assessment was conducted in a quiet room under low-level red light illumination. The rats displayed similar locomotion and rearing scores to one another and appeared to habituate to the open field arena by the second day of observation.

### Morris water maze training in rats

On postnatal day 80, animals were trained in the Morris water maze spatial learning task. Briefly, the water maze apparatus consists of a large circular pool (150 cm diameter, 80 cm deep) and a hidden platform (11 cm diameter). Both are constructed from black polyvinyl plastic, offering no intra-maze visual cues that may help guide escape behaviour. The platform was submerged 1.5 cm below the water's surface (temperature 26±1°C) and positioned 30 cm from the edge of the maze wall. The platform remained in the same position throughout the training session. The experimental room contained several extra-maze visual cues. The rat was lowered into the water facing the wall of the maze (30 cm high) at one of three locations which were alternated with each trial. Trials lasted a maximum of 90 s and the length of time taken for the rat to find the hidden platform was recorded. Rats failing to find the platform within the 90 s were placed on it for 10 s and allowed orientate themselves. The training session consisted of 5 trials with an inter-trial interval of 300 s. Each trained animal was assigned a corresponding passive control partner that spent the same lengths of time swimming in the pool, minus the platform. The latencies to find the maze platform were recorded for each trained rat over the 5-trial session (n = 4 trained rats). Latency-to-platform times decreased significantly (two-way ANOVA, Bonferroni post-tests, p<0.001) over the 5 trials of the training session indicating that the animals learned the task. After training, the rats were dried-off and placed back into their home cages. This was designated ‘Time 0 min’ after training. They were then sacrificed 30 min later, without anaesthetic, by cervical dislocation (n = 4 trained and passive controls, i.e. 8 animals in total). Following decapitation, their brains were quickly dissected out, covered in OCT and snap frozen in CO_2_-cooled n-hexane.

### Cryosectioning and immunofluorescence of Morris water maze-trained rat brains

Whole brains were cryosectioned coronally at −3.3 mm with respect to Bregma in order to reveal the dorsal hippocampus [53]. 12 µm sections were adhered to glass slides coated with poly-L-lysine. Sections were permeabilized using a solution of 0.1% Triton X in PBS for 25 min. Following this, sections were fixed in 70% ethanol for 25 min. The sections were then incubated overnight (18 h) with the primary antibody MAB3026 (Chemicon), which binds to the activated form of NF-κB. Following two 10 min washes in PBS, sections were incubated for 3 h with an anti-mouse secondary antibody labelled with the fluorescent marker FITC (Sigma). The secondary antibody was then washed off and sections were incubated in a solution of propidium iodide (PI) in order to stain all nuclei. A drop of Citifluor glycerol PBS solution (Agar Scientific) was used to mount each section with a coverslip and the slides were stored in darkness at 4°C.

### Confocal microscopy

All confocal images used for quantitative analysis of NF-κB activity (12-bit; 1024×1024 pixels) were captured using a 40X/0.8W water-immersion lens (Zeiss Achroplan). Images taken from Morris water maze-trained animals were captured from three defined regions of the hippocampus, i.e. CA1, CA3, and the apex of the dentate gyrus. The specific areas of the hippocampus that were imaged were kept consistent between sections. Confocal images of organotypic hippocampal cultures were captured from two distinct areas of the CA1, CA3 and dentate gyrus (i.e. 6 images per slice). Calcium imaging time-series frames (12-bit; 512×512 pixels) were captured using the 10X/0.3W Ph1 water-immersion lens (Zeiss Achroplan).

### Image and data analysis

Image analysis was conducted using the software package EBImage (http://www.bioconductor.org/packages/2.12/bioc/html/EBImage.html) for R statistical programming environment. Briefly, taking the immunofluorescently-labelled sections from water maze-trained animals as an example, the red and green channels were first separated for each image. Every pixel (1024×1024) was assigned an intensity value between 0 and 1. Using the red channel as a reference, size and fluorescence intensity thresholds were set in order to select only propidium iodide-labelled nuclei. A distance map was then generated for the image which calculates the distance each pixel is from an edge pixel. The watershed segmentation algorithm is then employed which accurately separates clusters of nuclei that are very close together, or touching, into individual cells. Minimum distance between objects and minimum radius criteria are written into the analysis scripts which further refines object separation. NF-κB activation was measured in the nucleus of every cell and then the average nuclear fluorescence intensity for NF-κB was calculated for the CA1, CA3 and dentate gyrus.

Calcium imaging time-series data was analysed using EBImage and individual cells were detected using similar object detection criteria described above. Baseline calcium fluorescence (F_0_) for each cell was taken as the average fluorescence intensity of that cell over the first 20 images, i.e. before TBS. The relative change in calcium fluorescence for every cell at increasing time points was then calculated using the equation ΔF/F_0_, where ΔF = [fluorescence at time X – baseline fluorescence (i.e. F_0_)]. The XY coordinates of each cell were used to calculate their distances from the electrode tip. Calcium response curves were smoothed via MATLAB (The MathWorks Inc.) inbuilt functions. Briefly, a moving average filter spanning 9 data points was used to reduce noise in the fluorescence time-course data. Non-responsive cells were excluded from the analysis (5,414 ‘non-responders’ in total). Non-responsive cells were defined as any cell that either achieved its peak calcium fluorescence prior to TBS, or any cell in which the difference between its maximum peak fluorescence signal and the mean of its signal was less than 0.25. To find the major peaks each calcium curve was processed by the MATLAB ‘peak finder routines’ (i.e. iPeak, developed by T. O'Haver: http://terpconnect.umd.edu/~toh/spectrum/PeakFindingandMeasurement.htm). Small local oscillations occurring near each major peak were neglected by selecting a time interval of 5 sec as the ‘minimum peak width’ and choosing an appropriate smoothing algorithm available in the options section of the ‘iPeak’ function. [Ca^2+^]_i_ oscillation frequency was calculated on a cell-by-cell basis by converting the ‘distance between peaks’ to millihertz (mHz).

## Results

### Theta-burst stimulation induces a radial-propagating calcium wave in the hippocampal network

We applied the theta-burst stimulation (TBS) protocol, described in figure 1A, to the molecular layer of the dentate gyrus of organotypic hippocampal slice cultures (figure 1B). The TBS electrical stimulus produced a slow-propagating calcium wave that spread radially from the electrode tip (figure 1C). We stimulated 15 slices in this way and, using automated image analysis, measured the average speed of calcium wave propagation to be 5.2±1 µm s^−1^. Figure 1D shows the spatiotemporal evolution of the changes in [Ca^2+^]_i_ after TBS is applied to one hippocampal slice. An exponential decrease in the peak [Ca^2+^]_i_ with increasing distance from the point of stimulation was observed.

**Figure 2:**
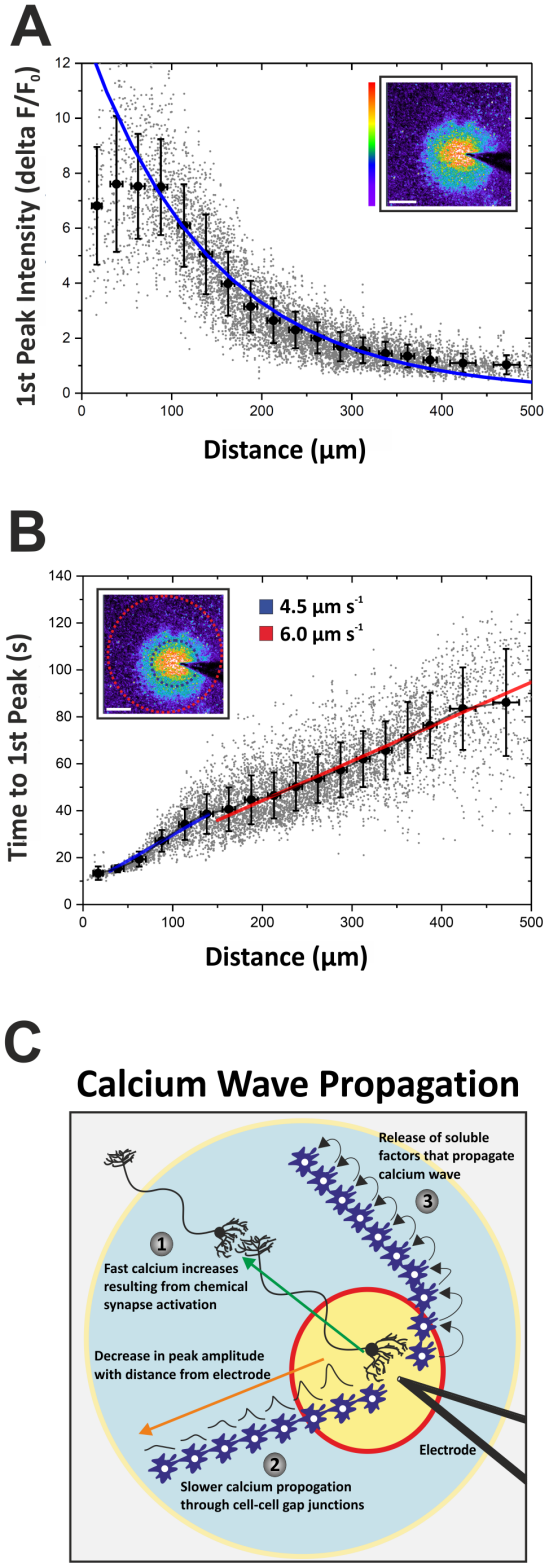
Calcium wave propagation in hippocampal slice cultures post-TBS. (A) Describes the relative increase in [Ca^2+^]_i_ concentration (i.e. 1^st^ Peak Intensity) in every cell (n = 6,536) post-TBS, with respect to distance from the electrode (0–500 µm). Within the first 100 µm from the point of stimulation, the Ca^2+^ fluorescence intensity of the first peak displays a wide range of values. From 100–500 µm, however, the fluorescence intensity of the first peak follows an exponential decay rate. Inset: Relative increase in [Ca^2+^]_i_ fluorescence, depicted by a pseudo-colour rainbow palette, in hippocampal cells 15 s after TBS termination. (B) Graphed is the time it takes each cell to achieve its first peak in [Ca^2+^]_i_ versus the distance that cell is from the electrode. Cells located further from the electrode take a longer time to reach their first peak in [Ca^2+^]_i_; the calcium wave travels at an average velocity of 5.2 ± 1 µm s^−1^. However, there appears to be a bi-phasic response in calcium wave propagation, illustrated by the blue and red linear fits of the data. The calcium wave travels slower 0–150 µm from the electrode, with an average speed of 4.5 µm s^−1^ (blue linear fit). Cells that lie 150–500 µm from the point of stimulation reach their first peak in [Ca^2+^]_i_ relatively faster (red linear fit) as the velocity of the calcium wave increases, reaching 6.0 µm s^−1^. Inset: Illustrates how the velocity of calcium wave propagation increases >150 µm from the electrode. (C) Schematic diagram describing the mechanisms by which changes in [Ca^2+^]_i_ may propagate over long distances in hippocampal slice cultures. The first method (1) is through action potential-mediated release of excitatory neurotransmitter (e.g. glutamate) at synapses located large distances from TBS-stimulated dendrites and cell bodies. This may explain the relatively small number of cells in panel B that are 300–500 µm from the electrode tip but exhibit very fast increases in [Ca^2+^]_i_ and ‘time-to-first peak’ responses. Because the velocity of calcium wave propagation in hippocampal slice cultures appears to increase >150 µm from the point of stimulation, this may represent a transition from (2) gap junction-mediated [Ca^2+^]_i_ propagation to (3) release of soluble factors (such as ATP) that stimulate neighbouring cells and perpetuate the calcium wave over long distances through astrocyte networks.

Next, we calculated the changes in calcium fluorescence over time for every cell positioned less than 500 µm from the electrode. Cells that did not exhibit significant calcium elevations were excluded from the analysis. As expected, cells located within 100 µm of the stimulation point displayed relatively large increases in [Ca^2+^]_i_ (figure 2A). Further than 150 µm from the electrode, however, the primary peak calcium signal was lower and decayed exponentially with increasing distance from the stimulation site (figure 2A). Cells located closer to the electrode, not surprisingly, achieved their first peak in [Ca^2+^]_i_ sooner than more distally-located cells. Interestingly, however, cells located within 150 µm of the electrode displayed a slower ‘time-to-peak’ [Ca^2+^]_i_ response after correcting for their absolute distance from the stimulation site. This is illustrated in figure 2B by the steeper slope of the blue linear fit (4.5 µm s^−1^; 0–150 µm), compared to the shallower slope of red linear fit (6.0 µm s^−1^; 150–500 µm), in the scatter plot. Cells located within 150 µm of the electrode achieved greater elevations in [Ca^2+^]_i_ post-TBS and, because of this, take slightly longer to achieve their peak calcium response. There were also notable differences in the ‘return-to-baseline’ kinetics of [Ca^2+^]_i_ at increasing distances from the electrode. The majority of cells exhibited semi-exponential decay rates after an initial peak in calcium, whereas a small proportion of cells (<1%) situated within 60 µm of the stimulation site, displayed linear decay constants (data not shown). This may indicate that different calcium-dependent signalling cascades are activated in a small number of cells very close to the TBS stimulus.

### Cells displaying large primary peaks in [Ca^2+^]_i_ have a delayed 2^nd^ peak

**Figure 3:**
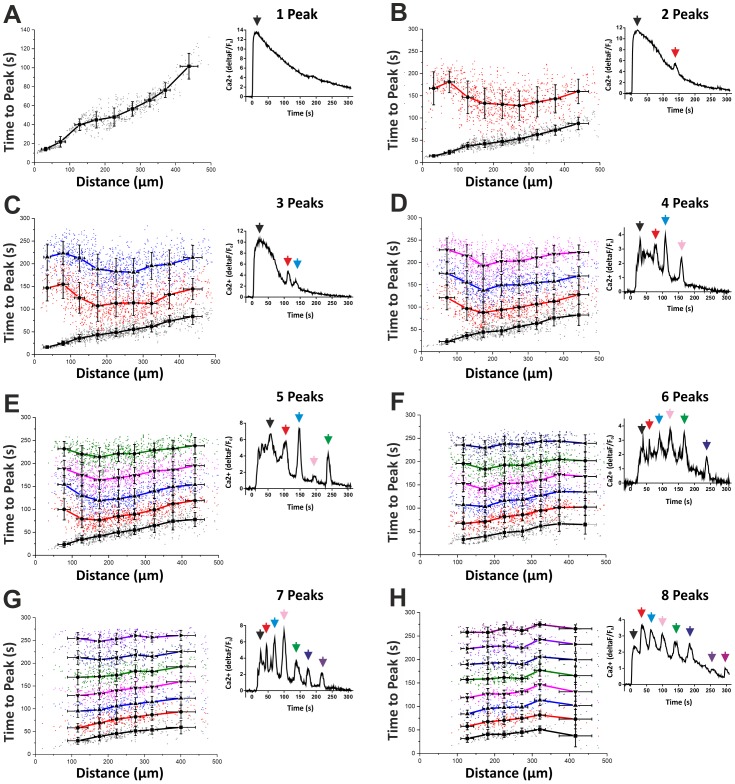
Time-to-peak for cells displaying multiple fluctuations in [Ca^2+^]_i_. (A) Time-to-peak versus distance from electrode for cells displaying one peak in [Ca^2+^]_i_. Inset: Relative changes in [Ca^2+^]_i_ over time for a single cell displaying one peak post-TBS. (B) Black line represents the ‘time-to-first peak’ in [Ca^2+^]_i_ for cells displaying two peaks. Red line represents the ‘time-to-second peak’. Notice the long delay in the second peak in cells within 150 µm from the point of stimulation. Therefore, cells with large first peaks in [Ca^2+^]_i_ take a long time to display a second peak. Cells located >300 µm from the electrode, however, display shorter durations between peak one and two. Inset: Relative changes in [Ca^2+^]_i_ over time post-TBS for a single cell displaying two peaks. (C) Black line represents the ‘time-to-first peak’ in [Ca^2+^]_i_ for cells displaying three peaks. Red and blue lines represent the ‘time-to-second and -third peaks’, respectively. Notice again the relatively large time between peak one and two in cells located <150 µm from the electrode. The time between second and third peaks is shorter, however. (D) Cells displaying four peaks in [Ca^2+^]_i_ post-TBS. Notice the regular interval between peaks 2, 3 and 4 (red, blue and magenta lines, respectively). (E–H) Time-to-peak versus distance from electrode for cells displaying 5, 6, 7 and 8 peaks in [Ca^2+^]_i_.

Almost all of the cells situated within 500 µm of the electrode achieved their primary peak in calcium within 120 s from the start of TBS (Figure 2B). Figure 3A graphs the ‘time-to-peak’ with respect to distance from the electrode for cells that possess only one peak. However, a large proportion of the total number of cells analysed (>85%) displayed multiple peaks in [Ca^2+^]_i_ following TBS. Therefore, we measured the ‘time-to-second peak’ (and also times to 3^rd^, 4^th^, 5^th^, 6^th^, 7^th^ and 8^th^ peaks) for all of these cells. Interestingly, the ‘time-to-second peak’, for cells displaying 2, 3 or 4 fluctuations in [Ca^2+^]_i_ and situated within 150 µm of the electrode, is delayed compared to more distal cells (figure 3B, C and D). This means that strongly stimulated cells close to the electrode display large first peaks in [Ca^2+^]_i_ and, therefore, require a longer latency period before a second spike in calcium can occur (figure 3B). In other words, the [Ca^2+^]_i_ has to drop to a certain level before a second peak can take place within the cell. This suggests that cells located further from the electrode, which display relatively modest primary peaks in [Ca^2+^]_i_, are more likely to begin oscillating sooner than cells located close to the electrode.

### Theta-burst stimulation induces calcium oscillations in hippocampal cells

**Figure 4:**
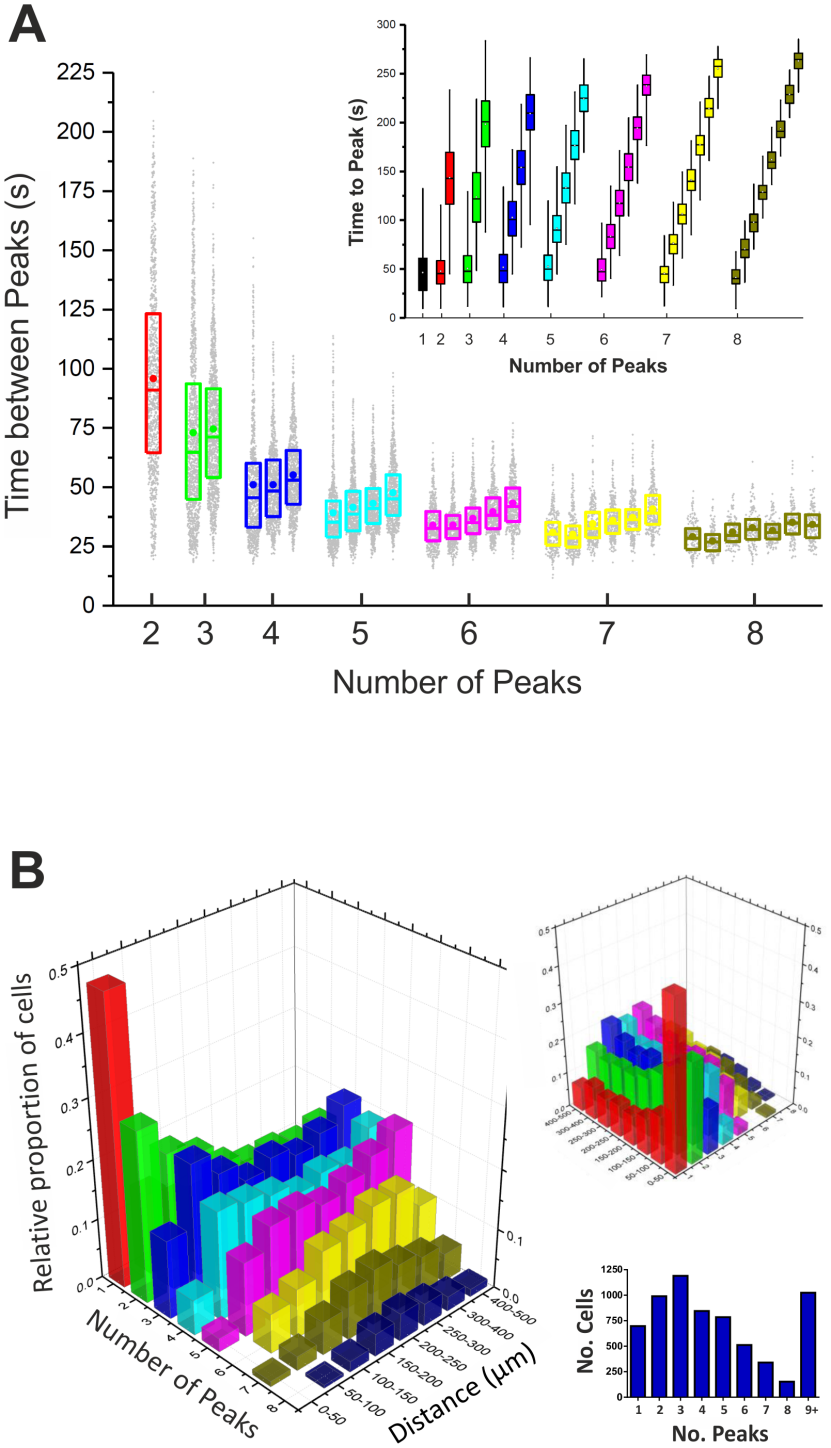
Calcium oscillation frequency with respect to distance from the point of TBS stimulation (A) Graphed is the ‘time-between-peaks’ for cells displaying between 2–8 fluctuations in [Ca^2+^]_i_. For cells containing either 2 or 3 peaks, there is large variability in the ‘time-between-peaks’. Cells with 4 peaks or greater, however, show regular frequencies of oscillations in [Ca^2+^]_i_ between approx. 22–33 mHz. Inset: Graphed is the ‘time-to-peak’ for cells displaying between 1–8 peaks in [Ca^2+^]_i_. (B) 3D graph representing the frequency of [Ca^2+^]_i_ oscillations in cells post-TBS with respect to their distance from the stimulation site. Plotted on the X axis is the ‘number of peaks’ each cell displays. The Y axis represents the distance the cell is from the electrode. Graphed on the Z axis is the relative proportion of cells with ‘n = 1–8 peaks’ at a given distance from the electrode. The graph shows that almost 50% of cells with one peak in [Ca^2+^]_i_ lie within 50 µm from the electrode. The further a cell is from the point of stimulation, the more likely it is to display multiple peaks in [Ca^2+^]_i_. Inset: 1,194 cells displayed 3 peaks in [Ca^2+^]_i_ post-TBS, the most common number of peaks; whereas 153 cells displayed 8 peaks. The total number of cells analysed was 6,536 and of these, 5,512 exhibited 1–8 peaks in [Ca^2+^]_i_ with the rest exhibiting greater than 8.

A striking result, clearly illustrated by figure 3, is that once a cell is stimulated with TBS and starts to display multiple oscillations in intracellular calcium, the time between subsequent peaks, i.e. peaks 2–3, peaks 3–4, peaks 4–5 etc., are remarkably regular. We analysed the frequencies of oscillations (i.e. ‘time between peaks’) in cells displaying up to 8 peaks in [Ca^2+^]_i_ (figure 3). Figure 4A summarises this analysis and charts the ‘time between peaks’ with respect to the number of peaks for each and every cell. Cells that exhibited 3 oscillations in [Ca^2+^]_i_ post-TBS did so at an average frequency of 17±8 mHz. For cells with 4 peaks, the frequency of [Ca^2+^]_i_ oscillations was 22±8 mHz. Cells displaying 5, 6, 7 or 8 peaks had corresponding frequencies of oscillation of 26±8 mHz, 29±8 mHz, 30±7 mHz or 33±7 mHz, respectively (figure 4A). Therefore, TBS induces large primary increases in [Ca^2+^]_i_ and also triggers calcium oscillations over a range of frequencies within stimulated cells. Moreover, our results indicate that the rhythm of oscillations, as determined by the ‘time-between-peaks’, was non-stochastic in nature and was strongly correlated with the cell's distance from the origin of electrical stimulation. This suggests that theta-burst stimulation induces a calcium wave that propagates large distances throughout the hippocampal neural network and may have far-reaching modulatory effects on synaptic plasticity.

### Calcium oscillations are more likely to occur in cells located >100 µm from the point of stimulation

Next, we calculated the proportion of cells displaying 1–8 peaks with respect to their distance from the point of stimulation (figure 4B). Almost 50% of cells possessing just one peak lay within 50 µm of the electrode. Moreover, less than 5% of the cells within the first 50 µm exhibited 5 or more intracellular calcium oscillations. In fact, the further the cell was from the electrode, the more likely it was to display multiple calcium oscillations (figure 4B). The most common number of oscillations in intracellular calcium post-TBS was 3, with 1194 (18%) cells exhibiting 3 peaks (figure 4B, inset). Over 50% of cells exhibited 4 peaks or more in the 5 min post-tetanic period, highlighting the widespread prevalence of [Ca^2+^]_i_ oscillations following this form of repetitive synaptic stimulation. Having assessed the effects of TBS on calcium dynamics, our next aim was to investigate a possible functional consequence of the observed [Ca^2+^]_i_ oscillations post-TBS in terms of what is already known about the molecular underpinnings of LTP induction and maintenance in the hippocampus. Therefore, we measured changes in the glutamate-inducible and [Ca^2+^]_i_-responsive transcription factor, NF-κB, after theta-burst stimulation.

### TBS induces NF-κB activity in CA1 pyramidal cells of organotypic hippocampal cultures

**Figure 5:**
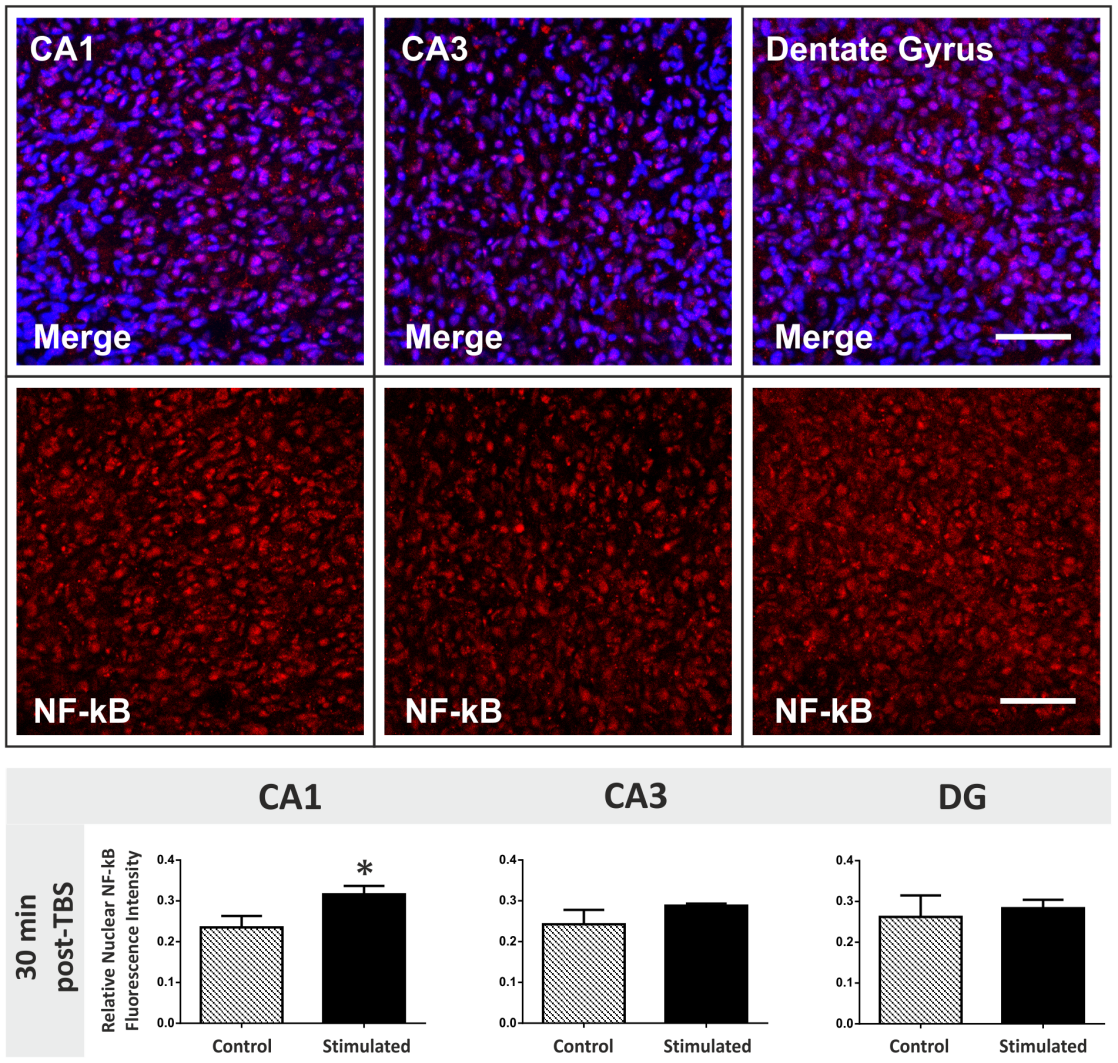
Changes in nuclear NF-κB levels 30 min post-TBS in the hippocampus. Top panel: NF-κB was immunofluorescently labelled in organotypic hippocampal slice cultures. The p65 subunit of activated NF-κB was stained red using a Cy3 secondary antibody and nuclei were counterstained blue using Hoechst 33258. High magnification confocal images (40X) of the CA1, CA3 and dentate gyrus neuronal cell populations were captured for each slice. Scale bar = 50 µm. Bottom graphs: Levels of activated nuclear NF-κB fluorescence were measured in organotypic hippocampal slices 30 min after theta-burst stimulation (TBS). CA1, CA3 and dentate gyrus cell populations were compared for control and stimulated slices. There is an up-regulation in activated nuclear NF-κB in the CA1 region (*two-tailed Student's t tests, p value<0.05).

Following theta-burst stimulation, organotypic hippocampal slices were fixed and stained for changes in the levels of activated nuclear NF-κB which could indicate the initiation of gene expression changes within stimulated cells. Changes in nuclear NF-κB levels were quantified in the CA1, CA3 and dentate gyrus 30 min post-TBS (figure 5). We found that NF-κB expression was significantly up-regulated in the CA1 region 30 min post-TBS. Nuclear NF-κB activation was unaltered in the CA3 and DG regions 30 min post-TBS, even though the stimulating electrode was positioned in the molecular layer of the DG. This suggests that theta-burst stimulation can activate transcription in cells located large distances from the stimulation site (CA1 >250 µm from electrode). This may, in part, be a consequence of the tri-synaptic feed-forward architecture of the hippocampal neuronal network. Strongly stimulated cells in the DG could transfer this information to the CA3 and onto CA1 neurons through activity-driven synaptic communication. Moreover, the TBS-induced slow-propagating calcium wave may also contribute to these long-range effects on NF-κB activation. The increased frequency of calcium oscillations in stimulated cells at greater distances from the electrode may explain NF-κB activation in the CA1 hippocampal region, but not dentate gyrus [47], [54]. Unfortunately, we were unable to retrospectively correlate TBS-induced calcium responses in individual cells with up-regulations in NF-κB activation specifically in the CA1 pyramidal cell layer.

### Spatial learning induces NF-κB activation in the CA1 region of the rat hippocampus

**Figure 6:**
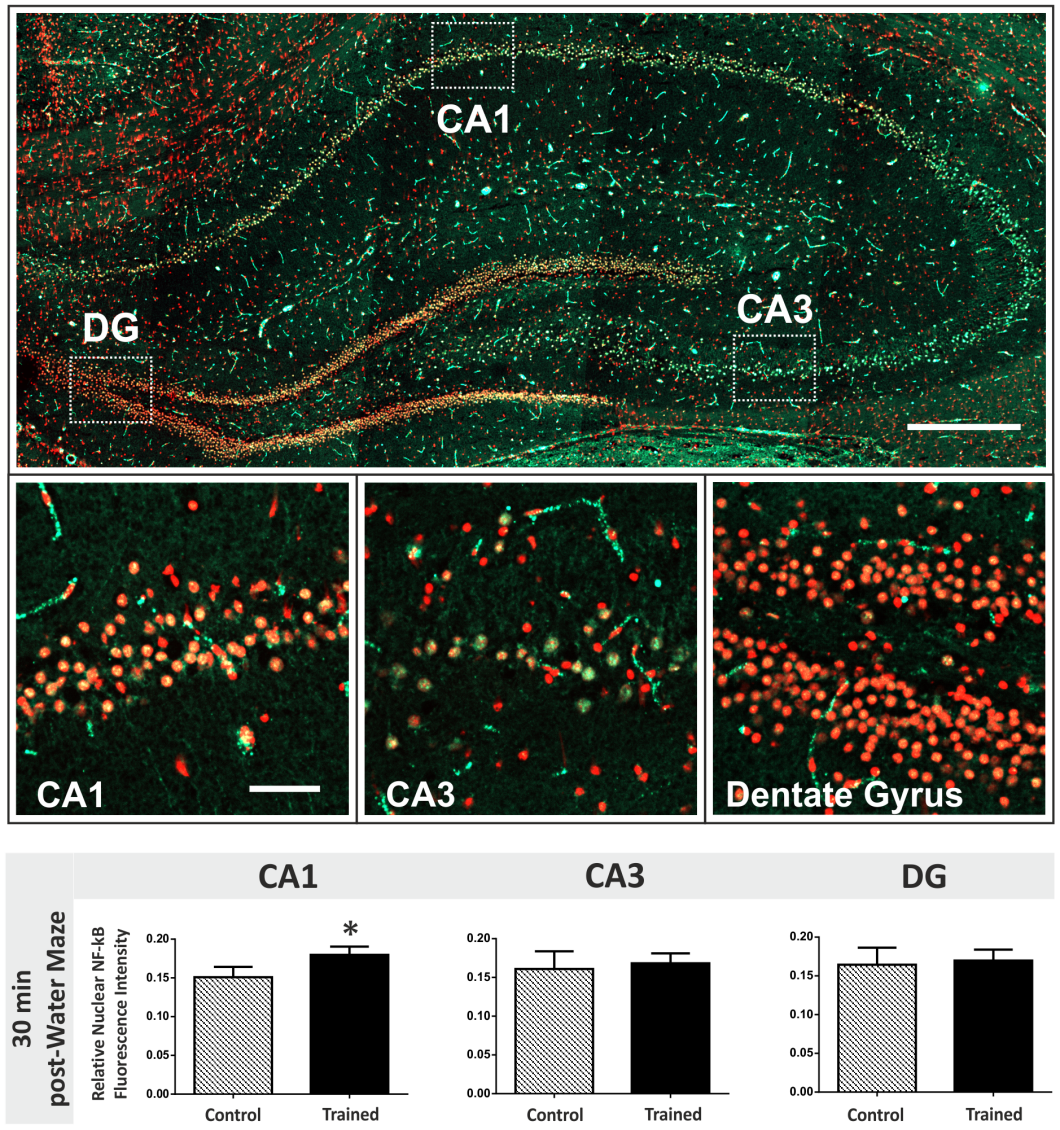
Changes in nuclear NF-κB levels 30 min post-spatial learning in the hippocampus. Top panel: Coronal section through the rat dorsal hippocampus (approx. −3.3 mm with respect to Bregma) immunofluorescently-labelled for the active form of the p65 subunit of NF-κB. Nuclei are stained red using propidium iodide and NF-κB labelled green using a FITC-conjugated secondary antibody. The white boxes represent the three regions of the hippocampal neuronal network that were analysed throughout this study. Scale bar = 400 µm. Middle panel: High-magnification images (40X) of the CA1, CA3 and DG illustrated by white boxes in the top montage. Scale bar = 50 µm. Bottom graphs: Levels of activated nuclear NF-κB fluorescence were measured in the rat hippocampus 30 min after water maze training ceased. CA1, CA3 and dentate gyrus cell populations were compared for passive control and trained rats. There is an up-regulation in activated nuclear NF-κB in the CA1 region (*two-tailed Student's t tests, p value<0.05).

We next investigated whether the activation of NF-κB measured in hippocampal slices following theta-burst stimulation is recapitulated *in vivo* in rats trained in the Morris water maze spatial learning task. Trained and passive control groups of rats were sacrificed 30 min post-training. Changes in nuclear NF-κB expression were quantified in the CA1, CA3 and dentate gyrus of the dorsal hippocampus; the area of the brain thought to be most important for spatial memory formation (figure 6). NF-κB activation was up-regulated specifically in the CA1 region 30 min post-water maze training. Therefore, similar cell type-specific changes in transcription factor activation in the hippocampus seem to underlie both *in vitro* theta-burst stimulation and spatial memory formation *in vivo*.

## Discussion

Calcium signalling in hippocampal neurons is fundamental to many processes including synaptic plasticity, long-term potentiation (LTP) and memory formation [2], [55]–[60]. Over the years, *in vitro* LTP studies in brain slices have reported a wide range of high-frequency stimulus (HFS) protocols used to model the cellular and molecular basis of learning and memory formation [61]. An important feature of the HFS protocol is the calcium response of directly stimulated, as well as interconnected, neurons and glial cells. In neuronal synapses, the magnitude of the calcium response is thought to determine whether the synapse will undergo a change in synaptic strength [62]. In astrocyte networks, the calcium response can influence the release of gliotransmitters which will have secondary modulatory effects on neuronal synapses in close proximity with glial cells [63]. In this study, we describe the propagating wave and oscillations in intracellular calcium [Ca^2+^]_i_ induced in a large proportion (>50%) of a transverse slice of the hippocampal network (an area of approx. 0.8 mm^2^) when a theta-burst stimulus (TBS) is applied to the dentate gyrus (DG) dendritic field. We recorded the simultaneous calcium response of hundreds of cells to TBS which is crucial if we are to even begin to understand how functionally connected neuronal ensembles translate a given stimulus into activity-driven changes in plasticity, gene expression and behaviour [64].

### TBS-induced [Ca^2+^]_i_ oscillations

Theta-burst stimulation mimics a physiologically-relevant frequency of neuronal activity exhibited in the hippocampus of behaving animals [29], [65]–[67]. By monitoring the dynamics of calcium fluctuations in response to TBS in a large proportion of the hippocampus we have uncovered a novel post-tetanic phenomenon; network-wide rhythmic calcium oscillations that range between 17–33 mHz in periodicity. Multiple [Ca^2+^]_i_ oscillations are more common in cells positioned further from the stimulation source (>100 µm). Cells located close to the electrode (<100 µm) experience a larger rise in [Ca^2+^]_i_ and it takes longer for the intracellular calcium levels to drop sufficiently (and/or intracellular stores to refill) before a second spontaneous rise in [Ca^2+^]_i_ can occur. This explains the large delay between first and second peaks in cells located <100 µm from the electrode. After the spontaneous second peak in [Ca^2+^]_i_ occurs, however, most cells appear to enter a post-tetanic phase of rhythmic [Ca^2+^]_i_ oscillations. It is likely that the [Ca^2+^]_i_ oscillations are heavily-dependent on calcium-induced calcium release (CICR) from intracellular stores [26], [27], [68], [69] and/or sustained extracellular calcium influx through store-operated calcium (SOC) channels that act to replenish calcium stores [70], [71]. [Ca^2+^]_i_ oscillations may also be functionally important in the bi-directional communication between neurons and astrocytes that accompanies TBS-induced changes in hippocampal synaptic plasticity [72], [73]. Since theta-burst stimulation mimics a physiologically-relevant frequency of neuronal activity in the hippocampus of behaving animals, we propose that these post-tetanic oscillations in [Ca^2+^]_i_ are functionally important for deciphering the information encoded in patterns of neuronal activation [72]. Moreover, the frequency of [Ca^2+^]_i_ oscillations triggered may be crucially important to the initial stimulus since the frequency increases with respect to distance from the stimulus origin. Cells situated within 100 µm of the electrode are likely to exhibit around 2 or 3 oscillations, whereas cells lying 400–500 µm from the stimulation point are more likely to display between 3–6 oscillations in [Ca^2+^]_i_. Therefore, the frequency of calcium oscillations in hippocampal cells post-TBS may be important in pattern separation, i.e. the ability of the hippocampus to process and code specific patterns of neuronal activity and to discriminate them from similar events. Therefore, since [Ca^2+^]_i_ oscillations exhibiting a frequency of 17–22 mHz are more likely to occur in cells located within 100 µm of the stimulation site and frequencies of 26–33 mHz are more common in cells situated 400–500 µm from the electrode, this information could facilitate the pattern separation needed for complex cognitive processes involving spatial learning and memory formation. Moreover, [Ca^2+^]_i_ oscillations of different frequencies are likely to activate distinct signalling cascades and changes in gene transcription within stimulated cells which could add a further layer of complexity to pattern separation and memory encoding in the hippocampus. In the present study, we report increased activation of the transcription factor, NF-κB, in the CA1 pyramidal cell layer of hippocampal slices 30 min after theta-burst stimulation to the dentate gyrus dendritic field. This cell-type specific translocation of activated NF-κB to the nucleus of CA1 cells may be functionally relevant for both LTP maintenance and memory formation since rats trained in the Morris water maze spatial learning task also displayed similar temporal and cell type-specific regulation of NF-κB activation.

### NF-κB and memory-associated synaptic plasticity

Since LTP is dependent on the synthesis of new proteins to maintain enhanced synaptic transmission at stimulated synapses [74], NF-κB activation in the CA1 region of hippocampal slices stimulated with TBS may indicate the transcription of genes important for LTP expression. NF-κB is reportedly activated both *in vitro* and *in vivo* in the hippocampus by high-frequency stimulation [48], [75] and is sensitive to both glutamate- and GABA-mediated synaptic transmission [76]. NF-κB is also present in synapses and can exhibit retrograde translocation to the nucleus upon activation [46], [77], [78]. Thus, as well as an activity-driven regulator of gene-expression, it is ubiquitously located within the cell and, therefore, ideally primed to act as a signalling molecule of glutamate-mediated calcium entry.

It is interesting that dentate granule cells display no alterations in NF-κB activation post-TBS. Either NF-κB up-regulation comes and goes within the first 30 min post-stimulation or dentate granule cells, being so close to the point of stimulation, are less likely to display multiple oscillations in [Ca^2+^]_i_ and, therefore, less NF-κB activation. Alternatively, NF-κB activation in the CA1 may not be a consequence of TBS-induced calcium oscillations. There are several studies, however, which do link [Ca^2+^]_i_ oscillations to NF-κB activity [79], [80]. It has also been shown that CaMKII activity is sensitive to [Ca^2+^]_i_ oscillations and CaMKII has been shown to regulate NF-κB activation [28], [44], [46], [81]. Consequently, if [Ca^2+^]_i_ oscillations are important for NF-κB activation, then DG cells, because of their close proximity to the electrode, may be less likely to exhibit up-regulations in NF-κB activation post-TBS. In addition to the propagating wave of calcium, the position of the electrode in the molecular layer of the dentate gyrus may directly stimulate some CA1 pyramidal neurons through axonal projections that synapse on dentate granule cells. The number of CA1 axons that synapse in the molecular layer of the dentate gyrus may also be exaggerated in organotypic slice cultures grown *in vitro* for 21 days since they tend to display more aberrant neuronal projections compared to acute hippocampal slices [82].

### TBS-induced calcium wave propagation

As well as serving to potentiate synaptic transmission between neurons, TBS initiates a wave of calcium within the interconnected astrocyte networks of the hippocampus. There are at least three main methods by which calcium waves can travel in an *in vitro* hippocampal slice culture (figure 2C). The predominant method, at least in acute hippocampal slices, is reportedly through stimulation-induced release of ATP and subsequent paracrine activation of purinergic receptors which perpetuate the release of ATP from neighbouring cells, thus initiating a calcium wave through astrocyte networks [83], [84]. Other signalling molecules, such as glutamate and nitric oxide, can also be released from astrocytes and contribute to calcium wave propagation [72], [85], [86]. The second is through cell-cell contacts and gap junctions which allow the movement of calcium ions and inositol 1,4,5-trisphosphate (IP3) into neighbouring cells, thus perpetuating the calcium wave over long distances [87], [88]. As the calcium wave travels further from the point of stimulation, the amplitude of the [Ca^2+^]_i_ response decays exponentially with distance (illustrated in figure 2C). A third way in which calcium waves propagate through the hippocampal network is mediated by action potential generation in neurons close to the electrode which synapse on other neurons and glial cells located up to hundreds of microns away from the site of stimulation [89]. This can explain the small number of cells in figure 2B, located 300–500 µm from the point of stimulation, which display relatively fast ‘time-to-first peak’ kinetics. Because the velocity of calcium wave propagation in hippocampal slice cultures appears to increase >150 µm from the point of stimulation, this may represent a transition from gap junction-mediated [Ca^2+^]_i_ propagation to release of soluble factors (such as ATP) that stimulate neighbouring cells and perpetuate the calcium wave over long distances through astrocyte networks. ATP is also a well-known trigger of [Ca^2+^]_i_ oscillations which could explain the increased number of oscillations at further distances from the point of stimulation.

The importance of astrocytes and other glial cells in hippocampal synaptic plasticity is becoming more and more evident in recent years [90]–[92]. Astrocytes can release neuromodulatory chemicals that can serve to either enhance or dampen synaptic transmission [93]–[97]. The triggering of calcium oscillations in astrocyte networks in the hippocampus could activate the release of neuromodulators from glial cells. Therefore, astrocytes may contribute to the decoding of the initial theta-burst stimulus and likely serve a pertinent role in LTP and synaptic plasticity changes in the hippocampus post-TBS stimulation [89], [98], [99].

### Conclusion

Here, we show that theta-burst stimulation, administered to the molecular layer of the dentate gyrus of hippocampal slices, induces similar temporal and cell-type specific activation of NF-κB to spatial learning in trained rats. This finding suggests that TBS in slice cultures may activate similar changes in gene expression that are important for memory formation in behaving animals. This confirms the validity and usefulness of this *in vitro* system to study the molecular basis of complex cognitive processes such as learning and memory formation. We have also uncovered a novel and interesting feature of theta-burst stimulation in the hippocampal network; the initiation of slow rhythmic [Ca^2+^]_i_ oscillations that may contribute to changes in synaptic plasticity and NF-κB-mediated changes in gene expression important for LTP maintenance. Moreover, changes in the frequency of [Ca^2+^]_i_ oscillations with increasing distance from the stimulation source may be important in pattern separation in the hippocampal network.
